# Two Compensation Strategies for Optimal Estimation in Sensor Networks with Random Matrices, Time-Correlated Noises, Deception Attacks and Packet Losses

**DOI:** 10.3390/s22218505

**Published:** 2022-11-04

**Authors:** Raquel Caballero-Águila, Jun Hu, Josefa Linares-Pérez

**Affiliations:** 1 Department of Statistics and Operations Research, University of Jaén, Campus Las Lagunillas, 23071 Jaén, Spain; 2 Key Laboratory of Advanced Manufacturing and Intelligent Technology, Ministry of Education, Harbin University of Science and Technology, Harbin 150080, China; 3 Department of Statistics and Operations Research, University of Granada, Av. Fuentenueva, 18071 Granada, Spain

**Keywords:** centralized fusion estimation, random parameter matrices, time-correlated noise, deception attacks, packet dropouts

## Abstract

Due to its great importance in several applied and theoretical fields, the signal estimation problem in multisensor systems has grown into a significant research area. Networked systems are known to suffer random flaws, which, if not appropriately addressed, can deteriorate the performance of the estimators substantially. Thus, the development of estimation algorithms accounting for these random phenomena has received a lot of research attention. In this paper, the centralized fusion linear estimation problem is discussed under the assumption that the sensor measurements are affected by random parameter matrices, perturbed by time-correlated additive noises, exposed to random deception attacks and subject to random packet dropouts during transmission. A covariance-based methodology and two compensation strategies based on measurement prediction are used to design recursive filtering and fixed-point smoothing algorithms. The measurement differencing method—typically used to deal with the measurement noise time-correlation—is unsuccessful for these kinds of systems with packet losses because some sensor measurements are randomly lost and, consequently, cannot be processed. Therefore, we adopt an alternative approach based on the direct estimation of the measurement noises and the innovation technique. The two proposed compensation scenarios are contrasted through a simulation example, in which the effect of the different uncertainties on the estimation accuracy is also evaluated.

## 1. Introduction

As a fundamental topic in the fields of control and signal processing, the estimation problem in networked systems has attracted great research attention in recent years. The presence of different uncertainty sources—errors in the measurement devices, limitations in transmission processes or vulnerability of the network, among others—often causes certain limitations, such as lack of signal information (commonly referred to as uncertain or missing observations), fading measurements or transmission delays and packet dropouts, which are usually random in nature. The performance of the estimators proposed in conventional systems can significantly degrade due to these constraints, which typically result in random imperfections in the data available for estimation. As a result, new problems arise when studying the estimation problem in multisensor systems with networked-induced phenomena. A comprehensive review of the main results and new challenges related to this problem can be found in [1,2,3,4].

Some of the aforementioned networked-induced phenomena that occur in a great variety of application fields—e.g., digital control of chemical processes, radar control, navigation systems and economic systems—can be modeled by including stochastic parameters in the measurement equations. Consequently, the use of random parameter matrices in the mathematical model of the sensor measurements offers a unified framework for describing such random events. Systems with multiplicative noises are a specific example of systems with random measurement matrices and are of tremendous interest due to their applicability in various areas of communication, image processing, etc. Systems with uncertain observations or sensor gain degradation serve as another example. In addition, networked systems with random delays can be transformed into systems with random matrices. These facts, among others, explain why research on the estimation problem in these kinds of systems with random parameter matrices have become increasingly popular over the past years. For some representative contributions, see, for example, [5,6,7,8,9,10] and references therein.

Over the last few decades, the research on the estimation problem in networked systems with packet dropouts has been considerably reported. Random packet losses during the process of data transmission can be caused by congestion-related buffer overflows, transmission failures in the physical network links or long transmission delays that cause the discarding of outdated packets, among other reasons. A major topic in this kind of systems is how to compensate the data packets that are lost. The most popular compensation strategies are the zero-input mechanism and the hold-input mechanism, under which the filter input is either set to zero or it is held at the most recent data that were received, respectively, when the current data are lost [11,12,13,14]. In [15], a packet dropout compensation framework that includes the popular zero-input and hold-input mechanisms as special cases is proposed. An alternative prediction compensation methodology has drawn the attention of several authors in recent years; this methodology involves compensating each missed measurement packet by its predictor (see, e.g., [16,17,18] and references therein). Using this compensation strategy, centralized fusion estimators, including filter, predictor and smoother, in the linear unbiased minimum variance sense are designed in [16]. The problem of self-tuning distributed fusion state estimation in networked systems with unknown packet receiving rates, noise variances and model parameters is addressed in [17]. A solution to the distributed fusion estimation problem in systems with random parameter matrices is proposed in [18], under the assumption that transmissions to local processors may experience one-step delays and packet dropouts, and either one or two measurements can be simultaneously processed at each time instant.

In practice, time-correlated measurement and channel noises are found in many engineering applications, such as radar systems, global navigation satellite systems, or wireless networks, where the sampling frequency is usually high enough, thus making the measurement noises be significantly correlated in two or more consecutive sampling periods. The estimation problem under the assumption that the time-correlated measurement noises are the output of a linear system model with white noise has been an important research focus during the past years. The most popular methods to deal with this kind of noise correlation are the state augmentation method—which is simple and direct, but computationally expensive— and the measurement differencing method, which avoids increasing dimensions but needs two consecutive measurements to compute the difference (see, e.g., [19,20,21,22,23]). It should be noted that most of the published results are concerned with the estimation problem in single-sensor systems, but the fusion estimation problem in networked systems has received significantly less attention (see, e.g., [24,25]).

When the system is subject to random delays or packet dropouts, the sensor measurement may not be received on time at the processor, and consequently, the measurement differencing method cannot be used. In other words, for systems having packet losses, the measurement differencing method will not work, and finding new non-augmentation methods to deal with the time-correlated measurement noise in these kinds of systems is a challenging issue. The state estimation problem for stochastic uncertain systems with time-correlated additive noises and random packet dropouts in transmission is addressed in [26]—for linear systems—and [27]—for non-linear systems—using the predictor of a sensor measurement as compensation when such measurement is lost.

Despite their undeniable benefits, sensor networks have some weaknesses that must be considered when dealing with the estimation problem to guarantee the accuracy of the designed estimators. One of the most common dangers that make a network less reliable is the possibility of suffering cyber-attacks, and analyzing the success rate of such attacks launched by adversaries has recently become a research topic of great interest. In practice, successful attacks can usually be understood as intermittent or random in their implementation. A comprehensive literature review related to cyber-attacks on networked systems can be found in [28].

One of the most common types of attacks are the so-called deception attacks, which violate data integrity by purposefully altering sensor measurements. The complexity and significance of studying the estimation problem in networked systems subject to deception attacks have inspired many fruitful efforts by the scientific community. In [29], an integrated approach is proposed to simultaneously address the problems of detection and estimation in discrete-time stochastic systems with event-triggered transmission, subject to random disturbances and deception attacks. The problem of detection against deception attacks in a remote estimation framework in multi-sensor systems is addressed in [30]. The distributed filtering problem for discrete-time systems with multiplicative noises and deception attacks has been studied in [31]. In [32], a cluster-based approach is used to address the distributed fusion estimation problem for networked systems when measurements are subject to random deception attacks and the distributed estimation problem in networked systems with a given topology has been studied in [33]—under false data injection attacks—and in [34,35]—under deception attacks.

### Main Contributions and Related Work

To the best of our knowledge, the optimal linear estimation problem in multi-sensor systems with random parameter matrices and time-correlated additive noises has not been fully investigated when the measurements are subject to random deception attacks, and packet dropouts may occur during transmission. Motivated by the above discussion, we consider a system model with the following characteristics: (a) the evolution model of the signal to be estimated does not need to be known, since a covariance-based estimation approach is used; (b) the model for the sensor-measured output under consideration includes random parameter matrices, offering a broad framework for many network-induced phenomena; (c) the sensor measured outputs are randomly affected by deception attacks; (d) random packet dropouts may occur during data transmissions from the sensors to the processing center and two different compensation models—based on measurement prediction—are proposed to describe the measurements available after transmission. Apart from the fact of considering a widespread system model that covers many general situations, the most significant contribution of this paper lies in the fact that, in contrast to the existing state-augmentation and measurement differencing methods, a non-augmentation technique is used to deal with the time-correlation of the additive noises. More precisely, through the direct estimation of the measurement noises and using the innovation technique under a covariance-based approach, recursive centralized fusion filtering and fixed-point smoothing algorithms are designed.

**Figure 1:**
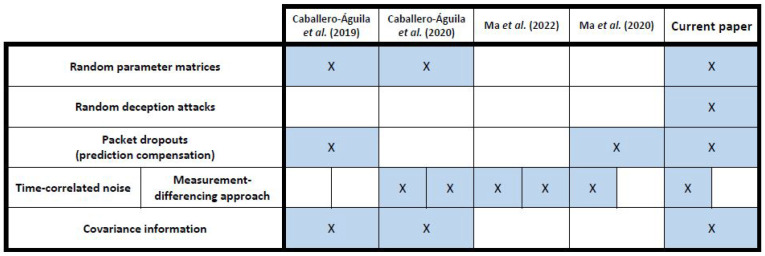
Related work [18,24,25,26]: comparison with the current paper.

Some of the most closely related papers in the literature are [18,24,25,26]. In [18], systems with random parameter matrices, one-step delays, packet dropouts and multi-packet processing are considered; the main difference between the system model in [18] and the current one is the presence of deception attacks and time-correlated noises, apart from the fact that [18] allows the possibility that two packets can be received at each instant of time. In [24], using the measurement-differencing method, centralized and distributed fusion filtering and fixed-point smoothing algorithms are designed for networked systems with random parameter matrices and time-correlated channel noise; so, in addition to the fact that we do not use the measurement-differencing approach, the key distinction with the current results resides in the presence of random deception attacks and the possibility of random packet dropouts in the transmission. The centralized and sequential fusion filtering problems for networked uncertain systems, where the measurement noises are time-correlated, are addressed in [25]; our study also considers time-correlated additive noises and it extends the results in [25] by including random parameter matrices and deception attacks in the measurement model, as well as random packet dropouts in the transmission. Finally, random packet dropouts in transmission and time-correlated additive noises are simultaneously considered in [26], but neither random parameter matrices nor random deception attacks are considered in the measurement equation. It is also noteworthy that the derivation in the estimation algorithms in [25,26] is based on the knowledge of the state-space model, whereas the algorithms proposed in the current paper do not require the signal evolution equation, but just the covariance function factorization into a separable form (covariance information). These considerations are summarized in Figure 1.

*Paper structure.* The paper’s structure is outlined as follows. The problem under consideration and the characteristics of the observation model are described in Section 2, with special emphasis on the two compensation strategies proposed. The main results are presented in Section 3, where some auxiliary lemmas are firstly introduced before the optimal linear filtering and smoothing estimation algorithms are derived under the two compensation frameworks considered. In Section 4, a simulation example is presented to show the feasibility of the proposed estimators. Finally, some conclusions are drawn in Section 5.

*Notation.* As far as possible, standard mathematical notation will be used. If not explicitly stated, all vectors and matrices are assumed to be of suitable dimensions, compatible with algebraic operations.
$R^{n}$ and $R^{m\times n}$Set of *n*-dimensional real vectors and set of m×n real matrices$\delta_{k,h}$Kronecker delta function$M^{T}$ and $M^{-1}$Transpose and inverse of matrix *M*$M^{(a)T}$ and $M^{(a)-1}$Shorthand for ${\left(M^{\left(a\right)}\right)}^{T}$ and ${\left(M^{\left(a\right)}\right)}^{-1}$$\left(M_{1},\ldots ,M_{k}\right)$Partitioned matrix whose blocks are the submatrices $M_{1},\ldots ,M_{k}$$Diag(N_{1},\ldots ,N_{m})$Block diagonal matrix, whose main-diagonal blocks are $N_{1},\ldots ,N_{m}$$I_{n}$ and $1_{n}$n×n identity matrix and n×1 all-ones vector0Zero scalar or matrix of compatible dimension⊗ and ∘Kronecker and Hadamard product of matrices, respectively$E\left[a\right]=\bar{a}$Mathematical expectation of a random vector or matrix *a*P(☆)Probability of an event ☆$\Sigma_{k,h}^{{ab}^{\left(ij\right)}}$Covariance of random vectors $a_{k}^{\left(i\right)}$ and $b_{h}^{\left(j\right)}$ ($\Sigma_{k,h}^{a^{\left(ij\right)}}=\Sigma_{k,h}^{{aa}^{\left(ij\right)}}$)
$\Sigma_{k,h}^{{ab}^{\left(ij\right)}}=Cov\left[a_{k}^{\left(i\right)},b_{h}^{\left(j\right)}\right]=E\left[\left(a_{k}^{\left(i\right)}-{\bar{a}}_{k}^{\left(i\right)}\right){\left(b_{h}^{\left(j\right)}-{\bar{b}}_{h}^{\left(j\right)}\right)}^{T}\right]$$G_{k}=G_{k,k}$Function $G_{k,h}$, depending on the time instants *k* and *h*, when h=k$F^{\left(i\right)}=F^{\left(ii\right)}$Function $F^{\left(ij\right)}$, depending on the sensors *i* and *j*, when i=j${\hat{a}}_{k/s}^{\left(*\right)}$Optimal linear estimator of the vector $a_{k}$ based on $\left\{y_{1}^{\left(*\right)},\ldots ,y_{s}^{\left(*\right)}\right\}$

## 2. Problem Statement and Observation Model

The goal of this work is to design recursive algorithms for the centralized fusion least-squares (LS) linear filtering and fixed-point smoothing problems, under the assumption that the sensor measurements of the signal to be estimated are transmitted over unreliable communication channels and random packet dropouts may occur during the process of data transmission. In addition, the output measurements—which are perturbed by time-correlated additive noises and subject to stochastic deception attacks—may randomly contain different uncertainties. By incorporating random parameter matrices into the measurement model, a general framework to model multiple random phenomena is proposed, including sensor gain degradation, missing or fading measurements, uncertainties brought on by the presence of multiplicative noise, or both multiplicative noises and missing measurements.

In order to compensate the lost data, two prediction compensation mechanisms are proposed, using either the predictor of the lost data packet, or the predictor of the data packet obtained by the sensor before the deception attack is launched.

### 2.1. Signal Process

The signal evolution equation does not need to be known, as the design of the proposed estimation algorithms will not be based on the state-space model—which requires an explicit mathematical model to express both the time variation of the signal and the relationship of that signal to the observations used for estimation. We will use a covariance-based estimation approach for the design of the algorithms that requires only the first- and second-order moments of the processes involved in the model describing the observations of all sensors. The advantage of this approach is that it is not necessary to derive a different estimation algorithm when the signal evolution model varies; instead, the mean function of the signal is assumed to be zero and its covariance function is expressed in a separable form. More specifically, the following assumption on the signal process is required:

**Hypothesis** **(H1).** *The signal ${\left\{x_{k}\right\}}_{k\geq 1}$ is an $n_{x}$-dimensional second-order zero-mean random process, whose covariance function can be factorized in a separable form: $\Sigma_{k,h}^{x}=E\left[x_{k}x_{h}^{T}\right]=A_{k}B_{h}^{T},\;h\leq k,$ where $A_{k},B_{h}\in R^{n_{x}\times n}$ are known matrices.*

**Remark** **1.** *The separable form of the signal covariance function required in assumption (H1) covers many practical situations. For instance, when the state-space model is available, $x_{k}=\Phi_{k-1}x_{k-1}+w_{k-1},\;k\geq 1$, assuming non-singular transition matrices and a white noise independent of the initial state, the covariance function of the signal can be expressed as $E\left[x_{k}x_{h}^{T}\right]=\Phi_{k,h}E\left[x_{h}x_{h}^{T}\right],\;h\leq k,$ where $\Phi_{k,h}=\Phi_{k-1}\ldots \Phi_{h}$, and assumption (H1) is fulfilled taking, for example, $A_{k}=\Phi_{k,0}$ and $B_{h}=E\left[x_{h}x_{h}^{T}\right]{\left(\Phi_{h,0}^{-1}\right)}^{T}$ (see, e.g., [24]). Likewise, for the state-space model with stationary signals, $x_{k}=\Phi x_{k-1}+w_{k-1},\;k\geq 1$, under the same assumptions of non-singularity and independence, the covariance function can be expressed as $E\left[x_{k}x_{h}^{T}\right]=\Phi^{k-h}E\left[x_{h}x_{h}^{T}\right],\;h\leq k;$ then, taking $A_{k}=\Phi^{k}$ and $B_{h}=E\left[x_{h}x_{h}^{T}\right]{\left(\Phi^{-h}\right)}^{T}$, assumption (H1) is clearly true. Hence, the separability assumption on the signal autocovariance function required in (H1) covers different types of stationary and non-stationary signals and, as a result, the estimation problem approach based on such hypothesis provides a unifying approach to obtain general algorithms that are applicable to a wide range of practical situations, regardless of whether or not the state-space model is fully known. Consequently, the covariance-based estimation approach provides us with a large variety of options for dealing with different signal models without the need to design specific algorithms for each one. The signal of linear systems or that of uncertain systems with state-dependent multiplicative noise are examples of processes satisfying (H1), as it will be shown in Section 4.*

### 2.2. Measurement Model with Random Parameter Matrices and Time-Correlated Additive Noises

The measured outputs of the discrete-time random signal $x_{k}$ are assumed to be perturbed by random parameter matrices and time-correlated additive noises, according to the following model:

(1) $$z_{k}^{\left(i\right)}=C_{k}^{\left(i\right)}x_{k}+v_{k}^{\left(i\right)},\;\;k\geq 1;\;\;\;\;i=1,\ldots ,m,$$

where $z_{k}^{\left(i\right)}\in R^{n_{z}}$ is the output measurement of the *i*-th sensor at time *k*. The following hypotheses about the matrices ${\left\{C_{k}^{\left(i\right)}\right\}}_{k\geq 1}$ and the additive noises ${\left\{v_{k}^{\left(i\right)}\right\}}_{k\geq 1}$ are required:

**Hypothesis** **(H2).** *${\left\{C_{k}^{\left(i\right)}\right\}}_{k\geq 1}$, i=1,…,m, are independent sequences of independent random parameter matrices. Denoting $c_{{}_{pq}}^{\left(i\right)}\left(k\right)$ the (p,q)-th entry of $C_{k}^{\left(i\right)}$, the first and second-order moments $E\left[c_{{}_{pq}}^{\left(i\right)}\left(k\right)\right]$ and $E\left[c_{{}_{pq}}^{\left(i\right)}\left(k\right)c_{{}_{p^{\prime }q^{\prime }}}^{\left(j\right)}\left(k\right)\right]$, for i,j=1,…,m, $p,p^{\prime }=1,\ldots ,n_{z}$ and $q,q^{\prime }=1,\ldots ,n_{x}$, are assumed to be known.*

From (H2), it is clear that the means ${\bar{C}}_{k}^{\left(i\right)}=E\left[C_{k}^{\left(i\right)}\right]$ are known and their (p,q)-th entries are $E\left[c_{{}_{pq}}^{\left(i\right)}\left(k\right)\right],\;p=1,\ldots ,n_{z},\;q=1,\ldots ,n_{x}.$ Moreover, for any deterministic matrix $G\in R^{n_{x}\times n_{x}}$, the (p,q)-th entry of $E[C_{k}^{\left(i\right)}GC_{k}^{(j)T}]$ is given by $\sum_{a=1}^{n_{x}}\sum_{b=1}^{n_{x}}E\left[c_{{}_{pa}}^{\left(i\right)}\left(k\right)c_{{}_{qb}}^{\left(j\right)}\left(k\right)\right]G_{ab}$.

**Hypothesis** **(H3).** *The measurement noises ${\left\{v_{k}^{\left(i\right)}\right\}}_{k\geq 0}$, i=1,…,m, are time-correlated sequences satisfying*

(2) $$v_{k}^{\left(i\right)}=D_{k-1}^{\left(i\right)}v_{k-1}^{\left(i\right)}+\xi_{k-1}^{\left(i\right)},\;\;k\geq 1;\;\;\;\;i=1,\ldots ,m,$$

*where ${\left\{D_{k}^{\left(i\right)}\right\}}_{k\geq 0}$ are given deterministic parameter matrices and the following assumptions are required:*

**Hypothesis** **(H3(a)).** *$v_{0}^{\left(i\right)}$, i=1,…,m, are zero-mean, second-order random vectors with known covariance matrices*: $E\left[v_{0}^{\left(i\right)}v_{0}^{(j)T}\right]=\Sigma_{0}^{v^{\left(ij\right)}},\;i,j=1,\ldots ,m.$

**Hypothesis** **(H3(b)).** *${\left\{\xi_{k}^{\left(i\right)}\right\}}_{k\geq 0}$, i=1,…,m, are zero-mean second-order white processes. They are independent of each other, except at the same time instant, with known covariance matrices*: $E\left[\xi_{k}^{\left(i\right)}\xi_{h}^{(j)T}\right]=\Sigma_{k}^{\xi^{\left(ij\right)}}\delta_{k,h},\;k,h\geq 0;\;i,j=1,\ldots ,m.$

### 2.3. Stochastic Deception Attacks

Let us consider that the sensor measured outputs are randomly perturbed by deception attacks that are launched by a potential adversary, who injects some false information involving two components: one that neutralizes the actual measurements and a noise component, which is the blurred deceptive information added by the adversary. Specifically, at the *i*-th sensor, i=1,…,m, the deception signal is modeled as

$${\overset{˘}{z}}_{k}^{\left(i\right)}=-z_{k}^{\left(i\right)}+w_{k}^{\left(i\right)},\;\;\;\;k\geq 1;\;\;\;\;i=1,\ldots ,m,$$

where the following assumption on the noises ${\left\{w_{k}^{\left(i\right)}\right\}}_{k\geq 1}$ is required:

**Hypothesis** **(H4).** *The noises ${\left\{w_{k}^{\left(i\right)}\right\}}_{k\geq 1}$, i=1,…,m, are zero-mean, second-order white processes. They are independent of each other, except at the same time instant, with known covariance matrices: $E\left[w_{k}^{\left(i\right)}w_{h}^{(j)T}\right]=\Sigma_{k}^{w^{\left(ij\right)}}\delta_{k,h},\;\;k,h\geq 1;\;\;\;\;i,j=1,\ldots ,m.$*

At every sensor *i* and every sampling time *k*, the deception attacks may randomly succeed or fail, which can be modeled by a Bernoulli random variable, $\lambda_{k}^{\left(i\right)}$, taking the value one, if the attack is successful, and the value zero, if it is unsuccessful. More precisely, the sensor measurements, ${\overset{˘}{y}}_{k}^{\left(i\right)}$, subject to random deception attacks, are modeled by:

$${\overset{˘}{y}}_{k}^{\left(i\right)}=z_{k}^{\left(i\right)}+\lambda_{k}^{\left(i\right)}{\overset{˘}{z}}_{k}^{\left(i\right)},\;\;\;\;k\geq 1;\;\;\;\;i=1,\ldots ,m,$$

or, equivalently,

(3) $${\overset{˘}{y}}_{k}^{\left(i\right)}=\left(1-\lambda_{k}^{\left(i\right)}\right)z_{k}^{\left(i\right)}+\lambda_{k}^{\left(i\right)}w_{k}^{\left(i\right)},\;\;\;\;k\geq 1;\;\;\;\;i=1,\ldots ,m.$$

Hence, a successful attack ($\lambda_{k}^{\left(i\right)}=1$) means that only the noise injected by the adversary, $w_{k}^{\left(i\right)}$, will be transmitted to the processing center, while an unsuccessful attack ($\lambda_{k}^{\left(i\right)}=0$) means that the actual measured output, $z_{k}^{\left(i\right)}$, remains unchanged and will be transmitted intact. The following assumption on these Bernoulli random variables is required:

**Hypothesis** **(H5).** *${\left\{\lambda_{k}^{\left(i\right)}\right\}}_{k\geq 1}$, i=1,…,m, are independent white sequences of Bernoulli random variables with known probabilities $P\left(\lambda_{k}^{\left(i\right)}=1\right)={\bar{\lambda }}_{k}^{\left(i\right)},\;k\geq 1.$*

### 2.4. Observation Model after Transmission: Two Packet Dropout Compensation Strategies

Let us consider that there exist random packet dropouts during data transmissions from the sensors to the processing center. This will be modeled by a Bernoulli random variable, $\gamma_{k}^{\left(i\right)}$, taking the value one, if the transmission is successful, and the value zero, if the corresponding data packet is lost during transmission. The following assumption on these Bernoulli random variables is required:

**Hypothesis** **(H6).** *${\left\{\gamma_{k}^{\left(i\right)}\right\}}_{k\geq 1}$, i=1,…,m, are independent white sequences of Bernoulli random variables with known probabilities $P\left(\gamma_{k}^{\left(i\right)}=1\right)={\bar{\gamma }}_{k}^{\left(i\right)},\;k\geq 1.$*

When the current measurement is not received at the processing center, a compensating measurement will be used instead. Among the different compensation strategies proposed in the literature, we focus on the prediction compensation approach. Taking into account that the observation model under consideration is subject to stochastic deception attacks, two possible compensation models naturally arise:

*Model I*: Compensation with the prediction estimator of the measurement that is lost (i.e., the one transmitted by the sensor after the attack is launched), ${\hat{\overset{˘}{y}}}_{k/k-1}^{(i)I}$. The observations used at the processing center in this case are given by:

(4) $$y_{k}^{(i)I}=\gamma_{k}^{\left(i\right)}{\overset{˘}{y}}_{k}^{\left(i\right)}+\left(1-\gamma_{k}^{\left(i\right)}\right){\hat{\overset{˘}{y}}}_{k/k-1}^{(i)I},\;\;k\geq 1;\;\;\;i=1,\ldots ,m.$$

*Model II*: Compensation with the prediction estimator of the actual sensor measured output (the one obtained by the sensor before the attack is launched), ${\hat{z}}_{k/k-1}^{(i)II}$. The observation model after transmission in this case is given by:

(5) $$y_{k}^{(i)I\;I}=\gamma_{k}^{\left(i\right)}{\overset{˘}{y}}_{k}^{\left(i\right)}+\left(1-\gamma_{k}^{\left(i\right)}\right){\hat{z}}_{k/k-1}^{(i)I\;I},\;\;k\geq 1;\;\;\;i=1,\ldots ,m.$$

Finally, the following independence assumption is required:

**Hypothesis** **(H7).** *For i=1,…,m, the signal process ${\left\{x_{k}\right\}}_{k\geq 1}$, the vector $v_{0}^{\left(i\right)}$ and the processes ${\left\{C_{k}^{\left(i\right)}\right\}}_{k\geq 1}$, ${\left\{\xi_{k}^{\left(i\right)}\right\}}_{k\geq 0}$, ${\left\{w_{k}^{\left(i\right)}\right\}}_{k\geq 1}$, ${\left\{\lambda_{k}^{\left(i\right)}\right\}}_{k\geq 1}$ and ${\left\{\gamma_{k}^{\left(i\right)}\right\}}_{k\geq 1}$ are mutually independent.*

**Remark** **2.** *The proposed compensation strategies work as follows:*

- *If $\gamma_{k}^{\left(i\right)}=1$ (successful transmission from sensor i with no loss at the sampling time k), ${\overset{˘}{y}}_{k}^{\left(i\right)}$ will be used for the estimation under both models.*
- *If $\gamma_{k}^{\left(i\right)}=0$ (at the sampling time k, the data packet from sensor i is lost):*
  - *The predictor of ${\overset{˘}{y}}_{k}^{\left(i\right)}$, given by ${\hat{\overset{˘}{y}}}_{k/k-1}^{(i)I}=\left(1-{\bar{\lambda }}_{k}^{\left(i\right)}\right){\hat{z}}_{k/k-1}^{(i)I}$, will be used for the estimation under Model I. Consequently, the compensating measurement considered is the predictor of $z_{k}^{\left(i\right)}$ weighted by the probability that $z_{k}^{\left(i\right)}$ has not been attacked at time k.*
  - *The predictor of $z_{k}^{\left(i\right)}$, that is ${\hat{z}}_{k/k-1}^{(i)I\;I}$, will be used for the estimation under Model II. Consequently, the compensating measurement considered in this case does not take into account the possibility of an attack.*

**Remark** **3.** *For the considered kind of systems with time-correlated additive noises and transmission random losses, the measurement differencing method—typically used to deal with the time-correlation phenomena—is not successful, since some sensor measurements are randomly lost and, consequently, cannot be processed (see [26]). Therefore, an alternative approach, based on the direct estimation of the measurement noises and the innovation technique, will be used to address the centralized fusion optimal linear estimation problem in the next section.*

## 3. Main Results

Our aim is to design centralized fusion optimal linear filtering and fixed-point smoothing algorithms, based on the observations within the compensation frameworks proposed in the previous section. For this purpose, at every sampling time, k≥1, we consider the vector constituted by the measurements of all sensors, $z_{k}={\left(z_{k}^{(1)T},\ldots ,z_{k}^{(m)T}\right)}^{T}$, satisfying the following stacked measurement equation, which is easily derived from (1):

(6) $$z_{k}=C_{k}x_{k}+v_{k},\;\;k\geq 1,$$

where $C_{k}={\left(C_{k}^{(1)T},\ldots ,C_{k}^{(m)T}\right)}^{T}\;\mathrm{and}\;v_{k}={\left(v_{k}^{(1)T},\ldots ,v_{k}^{(m)T}\right)}^{T}$. Let us observe that, from (2), the additive noise ${\left\{v_{k}\right\}}_{k\geq 0}$ is a time-correlated sequence, satisfying

(7) $$v_{k}=D_{k-1}v_{k-1}+\xi_{k-1},\;\;\;\;\;k\geq 1,$$

with $D_{k}=Diag\left(D_{k}^{\left(1\right)},\ldots ,D_{k}^{\left(m\right)}\right)$ and $\xi_{k}={\left(\xi_{k}^{(1)T},\ldots ,\xi_{k}^{(m)T}\right)}^{T}$.

Using (3) and denoting

$${\overset{˘}{y}}_{k}={\left({\overset{˘}{y}}_{k}^{(1)T},\ldots ,{\overset{˘}{y}}_{k}^{(m)T}\right)}^{T},\;\Lambda_{k}=Diag\left(\lambda_{k}^{\left(1\right)},\ldots ,\lambda_{k}^{\left(m\right)}\right)⊗I_{n_{z}}\;and\;w_{k}={\left(w_{k}^{(1)T},\ldots ,w_{k}^{(m)T}\right)}^{T},$$

it is straightforward to check that the stacked vector ${\overset{˘}{y}}_{k}$, constituted by the measurements subject to random deception attacks, satisfies the following equation:

(8) $${\overset{˘}{y}}_{k}=\left(I_{mn_{z}}-\Lambda_{k}\right)z_{k}+\Lambda_{k}w_{k},\;\;k\geq 1.$$

According to (4) and (5), and denoting

$$y_{k}^{\left(I\right)}={\left({\left(y_{k}^{(1)I}\right)}^{T},\ldots ,{\left(y_{k}^{(m)I}\right)}^{T}\right)}^{T},\;\;\;\;y_{k}^{\left(I\;I\right)}={\left({\left(y_{k}^{(1)I\;I}\right)}^{T},\ldots ,{\left(y_{k}^{(m)I\;I}\right)}^{T}\right)}^{T},$$

$${\hat{\overset{˘}{y}}}_{k/k-1}^{\left(I\right)}={\left({\left({\hat{\overset{˘}{y}}}_{k/k-1}^{(1)I}\right)}^{T},\ldots ,{\left({\hat{\overset{˘}{y}}}_{k/k-1}^{(m)I}\right)}^{T}\right)}^{T},\;\;\;\;{\hat{z}}_{k/k-1}^{\left(I\;I\right)}={\left({\left({\hat{z}}_{k/k-1}^{(1)I\;I}\right)}^{T},\ldots ,{\left({\hat{z}}_{k/k-1}^{(m)I\;I}\right)}^{T}\right)}^{T}$$

and $\Gamma_{k}=Diag\left(\gamma_{k}^{\left(1\right)},\ldots ,\gamma_{k}^{\left(m\right)}\right)⊗I_{n_{z}}$, the observations used for the estimation can be modeled by the following compact equations:

*Stacked Model I:*

(9) $$y_{k}^{\left(I\right)}=\Gamma_{k}{\overset{˘}{y}}_{k}+\left(I_{mn_{z}}-\Gamma_{k}\right){\hat{\overset{˘}{y}}}_{k/k-1}^{\left(I\right)},\;\;k\geq 1.$$

*Stacked Model II:*

(10) $$y_{k}^{\left(I\;I\right)}=\Gamma_{k}{\overset{˘}{y}}_{k}+\left(I_{mn_{z}}-\Gamma_{k}\right){\hat{z}}_{k/k-1}^{\left(I\;I\right)},\;\;k\geq 1.$$

Then, the centralized fusion optimal linear estimation (filtering and fixed-point smoothing) problem is reformulated as the task of finding the LS linear estimator of the signal, $x_{k}$, based on the observations up to the time instant k+N,N≥0, given in (9) or (10).

### 3.1. Preliminary Lemmas

Before deriving the centralized fusion filtering and fixed-point smoothing algorithms, we analyze the statistical properties of the processes involved in the observation models under consideration, as they will be necessary to address the LS linear estimation problem. These properties will be given in the following preliminary lemmas, whose proof is omitted since they follow quite easily from hypotheses (H1)–(H7).

**Lemma** **1.** *The processes involved in (6) and (7) satisfy the following properties:*

(a) *${\left\{C_{k}\right\}}_{k\geq 1}$ is a sequence of independent random parameter matrices with means ${\bar{C}}_{k}=E\left[C_{k}\right]={\left({\bar{C}}_{k}^{(1)T},\ldots ,{\bar{C}}_{k}^{(m)T}\right)}^{T},\;\;k\geq 1.$ Moreover, for any deterministic matrix $G\in R^{n_{x}\times n_{x}}$, the (i,j)-th entry of the matrix $E\left[C_{k}GC_{k}^{T}\right]$ is $E[C_{k}^{\left(i\right)}GC_{k}^{(j)T}],\;i,j=1,\ldots ,m.$*
(b) *$v_{0}$ is a zero-mean, second-order random vector with $\Sigma_{0}^{v}=E\left[v_{0}v_{0}^{T}\right]={\left(\Sigma_{0}^{v^{\left(ij\right)}}\right)}_{i,j=1,\ldots ,m}$.*
(c) *${\left\{\xi_{k}\right\}}_{k\geq 0}$ is a zero-mean, second-order white process with $\Sigma_{k}^{\xi }=E\left[\xi_{k}\xi_{k}^{T}\right]={\left(\Sigma_{k}^{\xi^{\left(ij\right)}}\right)}_{i,j=1,\ldots ,m}.$*
(d) *The covariance function of the time-correlated noise ${\left\{v_{k}\right\}}_{k\geq 0}$, $\Sigma_{k,h}^{v}=E\left[v_{k}v_{h}^{T}\right],\;h\leq k,$ admits the following factorization: $\Sigma_{k,h}^{v}=D_{k}F_{h}^{T}$, in which $D_{k}=D_{k,0},$$F_{h}^{T}=D_{h,0}^{-1}\Sigma_{h}^{v}$, $D_{k,h}=D_{k-1}\ldots D_{h}$ and $\Sigma_{h}^{v}$ is recursively computed as follows:*
$$\Sigma_{h}^{v}=D_{h-1}\Sigma_{h-1}^{v}D_{h-1}^{T}+\Sigma_{h-1}^{\xi },\;h\geq 1.$$

**Lemma** **2.** *The following properties hold for the processes involved in (8):*

(a) *${\left\{w_{k}\right\}}_{k\geq 1}$ is a zero-mean, second-order white process with $\Sigma_{k}^{w}=E\left[w_{k}w_{k}^{T}\right]={\left(\Sigma_{k}^{w^{\left(ij\right)}}\right)}_{i,j=1,\ldots ,m}.$*
(b) *${\left\{\Lambda_{k}\right\}}_{k\geq 1}$ is a sequence of independent random matrices with*
$${\bar{\Lambda }}_{k}=E\left[\Lambda_{k}\right]=Diag\left({\bar{\lambda }}_{k}^{\left(1\right)},\ldots ,{\bar{\lambda }}_{k}^{\left(m\right)}\right)⊗I_{n_{z}},\;\;k\geq 1.$$
*Denoting $\lambda_{k}={\left(\lambda_{k}^{\left(1\right)}1_{n_{z}}^{T},\ldots ,\lambda_{k}^{\left(m\right)}1_{n_{z}}^{T}\right)}^{T}={\left(\lambda_{k}^{\left(1\right)},\ldots ,\lambda_{k}^{\left(m\right)}\right)}^{T}⊗1_{n_{z}},\;k\geq 1$, the covariance matrices $K_{k}^{\lambda }=E\left[\lambda_{k}\lambda_{k}^{T}\right]$ and $K_{k}^{1-\lambda }=E\left[\left(1_{mn_{z}}-\lambda_{k}\right){\left(1_{mn_{z}}-\lambda_{k}\right)}^{T}\right]$ are known, and their entries can be computed taking into account that $E\left[\lambda_{k}^{\left(i\right)}\lambda_{k}^{\left(j\right)}\right]=\left\{\begin{array}{c} {\bar{\lambda }}_{k}^{\left(i\right)},\;\;i=j,\;\\ {\bar{\lambda }}_{k}^{\left(i\right)}{\bar{\lambda }}_{k}^{\left(j\right)},\;\;i\neq j. \end{array}\right.$*
(c) *The autocovariance function $\Sigma_{k}^{\overset{˘}{y}}=E\left[{\overset{˘}{y}}_{k}{\overset{˘}{y}}_{k}^{T}\right]$ is given by:*
(11) $$\Sigma_{k}^{\overset{˘}{y}}=K_{k}^{1-\lambda }\circ \left(E\left[C_{k}A_{k}B_{k}^{T}C_{k}^{T}\right]+D_{k}F_{k}^{T}\right)+K_{k}^{\lambda }\circ \Sigma_{k}^{w},\;\;k\geq 1.$$

**Lemma** **3.** *The stochastic process ${\left\{\Gamma_{k}\right\}}_{k\geq 1}$ modeling the packet dropout phenomena in the stacked observation models (9) and (10) is a sequence of independent random matrices with*

$${\bar{\Gamma }}_{k}=E\left[\Gamma_{k}\right]=Diag\left({\bar{\gamma }}_{k}^{\left(1\right)},\ldots ,{\bar{\gamma }}_{k}^{\left(m\right)}\right)⊗I_{n_{z}},\;\;\;k\geq 1.$$

*Denoting $\gamma_{k}={\left(\gamma_{k}^{\left(1\right)}1_{n_{z}}^{T},\ldots ,\gamma_{k}^{\left(m\right)}1_{n_{z}}^{T}\right)}^{T}={\left(\gamma_{k}^{\left(1\right)},\ldots ,\gamma_{k}^{\left(m\right)}\right)}^{T}⊗1_{n_{z}},\;k\geq 1$, the covariance matrices $K_{k}^{\gamma }=E\left[\gamma_{k}\gamma_{k}^{T}\right]$ are known, and their entries can be computed taking into account that $E\left[\gamma_{k}^{\left(i\right)}\gamma_{k}^{\left(j\right)}\right]=\left\{\begin{array}{c} {\bar{\gamma }}_{k}^{\left(i\right)},\;\;i=j,\\ {\bar{\gamma }}_{k}^{\left(i\right)}{\bar{\gamma }}_{k}^{\left(j\right)},\;\;i\neq j. \end{array}\right.$*

*Moreover, the signal process ${\left\{x_{k}\right\}}_{k\geq 1}$, the vector $v_{0}$ and the processes ${\left\{C_{k}\right\}}_{k\geq 1}$, ${\left\{\xi_{k}\right\}}_{k\geq 0}$, ${\left\{w_{k}\right\}}_{k\geq 1}$, ${\left\{\Lambda_{k}\right\}}_{k\geq 1}$ and ${\left\{\Gamma_{k}\right\}}_{k\geq 1}$ are mutually independent.*

### 3.2. Optimal Filtering and Fixed-Point Smoothing Algorithms (Model I)

We will use an innovation approach to obtain ${\hat{x}}_{k/L}^{\left(I\right)}$, the LS linear estimator of the signal $x_{k}$ based on the observations $\left\{y_{1}^{\left(I\right)},\ldots ,y_{L}^{\left(I\right)}\right\}$ defined by (9); more specifically, we aim at obtaining the signal filtering (L=k) and fixed-point smoothing (L=k+N,N≥1) estimators. According to this approach, the LS linear estimators of the signal, ${\hat{x}}_{k/L}^{\left(I\right)}$, based on a set of observations ${\left\{y_{h}^{\left(I\right)}\right\}}_{h\leq L}$, can be expressed as a linear combination of the innovations ${\left\{\mu_{h}^{\left(I\right)}\right\}}_{h\leq L}$ as follows:

(12) $${\hat{x}}_{k/L}^{\left(I\right)}=\sum_{h=1}^{L}X_{k,h}^{\left(I\right)}\Pi_{h}^{(I)-1}\mu_{h}^{\left(I\right)},\;\;k,L\geq 1,$$

where $X_{k,h}^{\left(I\right)}=E\left[x_{k}\mu_{h}^{(I)T}\right]$, and the innovations and their covariances are $\mu_{h}^{\left(I\right)}=y_{h}^{\left(I\right)}-{\hat{y}}_{h/h-1}^{\left(I\right)}$ and $\Pi_{h}^{\left(I\right)}=E\left[\mu_{h}^{\left(I\right)}\mu_{h}^{(I)T}\right]$, respectively.

So, the first key point is to find an appropriate expression for the one-stage observation predictors ${\hat{y}}_{h/h-1}^{\left(I\right)}$—or, equivalently, for the innovations $\mu_{h}^{\left(I\right)}$—that allows us to obtain the coefficients $X_{k,h}^{\left(I\right)}$ and the innovation covariances $\Pi_{h}^{\left(I\right)}$ appearing in expression (12) of ${\hat{x}}_{k/L}^{\left(I\right)}$.

From (9), taking into account the properties set out in Lemma 3 and the Orthogonal Projection Lemma (OPL), the observation predictors are given by:

$${\hat{y}}_{k/k-1}^{\left(I\right)}={\bar{\Gamma }}_{k}{\hat{\overset{˘}{y}}}_{k/k-1}^{\left(I\right)}+\left(I_{mn_{z}}-{\bar{\Gamma }}_{k}\right){\hat{\overset{˘}{y}}}_{k/k-1}^{\left(I\right)}={\hat{\overset{˘}{y}}}_{k/k-1}^{\left(I\right)},\;\;k\geq 1.$$

Using (8), Lemma 1 and the OPL, we obtain:

(13) $${\hat{\overset{˘}{y}}}_{k/k-1}^{\left(I\right)}=\left(I_{mn_{z}}-{\bar{\Lambda }}_{k}\right){\hat{z}}_{k/k-1}^{\left(I\right)},\;\;k\geq 1,$$

and from (6), Lemma 2 and using the OPL again, we obtain:

(14) $${\hat{z}}_{k/k-1}^{\left(I\right)}={\bar{C}}_{k}{\hat{x}}_{k/k-1}^{\left(I\right)}+{\hat{v}}_{k/k-1}^{\left(I\right)},\;\;k\geq 1.$$

Consequently, the innovations can be written as:

(15) $$\mu_{k}^{\left(I\right)}=\Gamma_{k}\left({\overset{˘}{y}}_{k}-\left(I_{mn_{z}}-{\bar{\Lambda }}_{k}\right)\left({\bar{C}}_{k}{\hat{x}}_{k/k-1}^{\left(I\right)}+{\hat{v}}_{k/k-1}^{\left(I\right)}\right)\right),\;\;k\geq 1.$$

Hence, we need the one-stage predictors of both the signal, ${\hat{x}}_{k/k-1}^{\left(I\right)}$, and the noise, ${\hat{v}}_{k/k-1}^{\left(I\right)}$. Note that, similarly to (12), defining now $V_{k,h}^{\left(I\right)}=E\left[v_{k}\mu_{h}^{(I)T}\right]$, the noise estimators, ${\hat{v}}_{k/L}^{\left(I\right)}$, are expressed as a linear combination of the innovations as follows:

(16) $${\hat{v}}_{k/L}^{\left(I\right)}=\sum_{h=1}^{L}V_{k,h}^{\left(I\right)}\Pi_{h}^{(I)-1}\mu_{h}^{\left(I\right)},\;\;k,L\geq 1.$$

Expressions (12) and (16) for the LS linear estimators as linear combination of the innovations, are the starting points to derive the recursive algorithms for the centralized LS linear filter, ${\hat{x}}_{k/k}^{\left(I\right)}$, and smoothers, ${\hat{x}}_{k/k+N}^{\left(I\right)}$, at the fixed point *k*, for N≥1, which are presented in the following theorem.

**Theorem** **1.** *Under hypotheses (H1) to (H7), the centralized LS linear filtering estimators, ${\hat{x}}_{k/k}^{\left(I\right)},$ and the corresponding error covariance matrices, ${\hat{\Sigma }}_{k/k}^{\left(I\right)}=E\left[\left(x_{k}-{\hat{x}}_{k/k}^{\left(I\right)}\right){\left(x_{k}-{\hat{x}}_{k/k}^{\left(I\right)}\right)}^{T}\right]$, are computed by:*

(17) $$\begin{array}{c} {\hat{x}}_{k/k}^{\left(I\right)}=\left(A_{k}\;,\;0\right)O_{k}^{\left(I\right)},\;\;k\geq 1,\\ {\hat{\Sigma }}_{k/k}^{\left(I\right)}=A_{k}B_{k}^{T}-\left(A_{k}\;,\;0\right)r_{k}^{\left(I\right)}{\left(A_{k}\;,\;0\right)}^{T},\;\;k\geq 1, \end{array}$$

*where*

(18) $$\begin{array}{c} O_{k}^{\left(I\right)}=O_{k-1}^{\left(I\right)}+J_{k}^{\left(I\right)}\Pi_{k}^{(I)-1}\mu_{k}^{\left(I\right)},\;\;k\geq 1;\;\;O_{0}^{\left(I\right)}=0,\\ J_{k}^{\left(I\right)}={\left(\left({\bar{C}}_{k}B_{k}\;,\;F_{k}\right)-\left({\bar{C}}_{k}A_{k}\;,\;D_{k}\right)r_{k-1}^{\left(I\right)}\right)}^{T}\left(I_{mn_{z}}-{\bar{\Lambda }}_{k}\right){\bar{\Gamma }}_{k},\;\;k\geq 1,\\ r_{k}^{\left(I\right)}=r_{k-1}^{\left(I\right)}+J_{k}^{\left(I\right)}\Pi_{k}^{(I)-1}J_{k}^{(I)T},\;\;k\geq 1;\;\;r_{0}^{\left(I\right)}=0. \end{array}$$

*The innovations, $\mu_{k}^{\left(I\right)}$, and their covariance matrices, $\Pi_{k}^{\left(I\right)}=E\left[\mu_{k}^{\left(I\right)}\mu_{k}^{(I)T}\right]$, are obtained by:*

(19) $$\begin{array}{c} \mu_{k}^{\left(I\right)}=y_{k}^{\left(I\right)}-\left(I_{mn_{z}}-{\bar{\Lambda }}_{h}\right)\left({\bar{C}}_{k}A_{k}\;,\;D_{k}\right)O_{k-1}^{\left(I\right)},\;\;k\geq 1,\\ \Pi_{k}^{\left(I\right)}=K_{k}^{\gamma }\circ \left(\Sigma_{k}^{\overset{˘}{y}}-\left(I_{mn_{z}}-{\bar{\Lambda }}_{k}\right)\Sigma_{k}^{{\hat{z}}^{\left(I\right)}}\left(I_{mn_{z}}-{\bar{\Lambda }}_{k}\right)\right),\;\;k\geq 1, \end{array}$$

*with $\Sigma_{k}^{\overset{˘}{y}}$ given in (11) and*

(20) $$\Sigma_{k}^{{\hat{z}}^{\left(I\right)}}=\left({\bar{C}}_{k}A_{k}\;,\;D_{k}\right)r_{k-1}^{\left(I\right)}{\left({\bar{C}}_{k}A_{k}\;,\;D_{k}\right)}^{T},\;\;k\geq 1.$$

*Additionally, at any sampling time k≥1, by starting from the filter, ${\hat{x}}_{k/k}^{\left(I\right)}$, and its error covariance matrix, ${\hat{\Sigma }}_{k/k}^{\left(I\right)}$, as initial conditions, the centralized LS linear smoothers, ${\hat{x}}_{k/k+N}^{\left(I\right)},$ and their error covariances, ${\hat{\Sigma }}_{k/k+N}^{\left(I\right)}=E\left[\left(x_{k}-{\hat{x}}_{k/k+N}^{\left(I\right)}\right){\left(x_{k}-{\hat{x}}_{k/k+N}^{\left(I\right)}\right)}^{T}\right]$, are recursively obtained as follows:*

(21) $$\begin{array}{c} {\hat{x}}_{k/k+N}^{\left(I\right)}={\hat{x}}_{k/k+N-1}^{\left(I\right)}+X_{k,k+N}^{\left(I\right)}\Pi_{k+N}^{(I)-1}\mu_{k+N}^{\left(I\right)},\;\;N\geq 1,\\ {\hat{\Sigma }}_{k/k+N}^{\left(I\right)}=\Sigma_{k/k+N-1}^{\left(I\right)}-X_{k,k+N}^{\left(I\right)}\Pi_{k+N}^{(I)-1}X_{k,k+N}^{(I)T},\;\;N\geq 1, \end{array}$$

*where $X_{k,k+N}^{\left(I\right)}=E\left[x_{k}\mu_{k+N}^{(I)T}\right]$ is computed by:*

(22) $$X_{k,k+N}^{\left(I\right)}=\left(\left(B_{k}\;,\;0\right)-M_{k,k+N-1}^{\left(I\right)}\right){\left({\bar{C}}_{k+N}A_{k+N}\;,\;D_{k+N}\right)}^{T}\left(I_{mn_{z}}-{\bar{\Lambda }}_{k+N}\right){\bar{\Gamma }}_{k+N},\;\;N\geq 1,$$

*and $M_{k,k+N}^{\left(I\right)}=E\left[{\hat{x}}_{k/k+N}^{\left(I\right)}O_{k+N}^{(I)T}\right]$ are obtained from the recursive formula*

(23) $$M_{k,k+N}^{\left(I\right)}=M_{k,k+N-1}^{\left(I\right)}+X_{k,k+N}^{\left(I\right)}\Pi_{k+N}^{(I)-1}J_{k+N}^{(I)T},\;\;N\geq 1;\;\;\;M_{k,k}^{\left(I\right)}=\left(A_{k}\;,\;0\right)r_{k}^{\left(I\right)}.$$

**Proof.**  We first obtain the signal prediction and filtering estimators ${\hat{x}}_{k/s}^{\left(I\right)},\;s\leq k$. From (15), it is clear that the coefficients $X_{k,h}^{\left(I\right)}=E\left[x_{k}\mu_{h}^{(I)T}\right]$ verify

$$X_{k,h}^{\left(I\right)}=\left(E\left[x_{k}{\overset{˘}{y}}_{h}^{T}\right]-\left(E\left[x_{k}{\hat{x}}_{h/h-1}^{(I)T}\right]{\bar{C}}_{h}^{T}+E\left[x_{k}{\hat{v}}_{h/h-1}^{(I)T}\right]\right)\left(I_{mn_{z}}-{\bar{\Lambda }}_{h}\right)\right){\bar{\Gamma }}_{h},\;\;1\leq h\leq k.$$

Now, using (8) and the properties of the processes involved in this equation, we have that $E\left[x_{k}{\overset{˘}{y}}_{h}^{T}\right]=A_{k}B_{h}^{T}{\bar{C}}_{h}^{T}\left(I_{mn_{z}}-{\bar{\Lambda }}_{h}\right)$, and from (12) and (16), we obtain

$$E\left[x_{k}{\hat{x}}_{h/h-1}^{(I)T}\right]=\sum_{j=1}^{h-1}X_{k,j}^{\left(I\right)}\Pi_{j}^{(I)-1}X_{h,j}^{(I)T};\;\;E\left[x_{k}{\hat{v}}_{h/h-1}^{(I)T}\right]=\sum_{j=1}^{h-1}X_{k,j}^{\left(I\right)}\Pi_{j}^{(I)-1}V_{h,j}^{(I)T},\;\;h\geq 2.$$

Hence,

$$X_{k,h}^{\left(I\right)}\;=\left[A_{k}B_{h}^{T}{\bar{C}}_{h}^{T}-\left(1-\delta_{h,1}\right)\sum_{j=1}^{h-1}X_{k,j}^{\left(I\right)}\Pi_{j}^{(I)-1}{\left({\bar{C}}_{h}X_{h,j}^{\left(I\right)}+V_{h,j}^{\left(I\right)}\right)}^{T}\right]\left(I_{mn_{z}}-{\bar{\Lambda }}_{h}\right){\bar{\Gamma }}_{h},\;\;\;1\leq h\leq k.$$

Then, by defining:

(24) $$J_{h}^{x(I)}=\left[B_{h}^{T}{\bar{C}}_{h}^{T}-\left(1-\delta_{h,1}\right)\sum_{j=1}^{h-1}J_{j}^{x(I)}\Pi_{j}^{(I)-1}{\left({\bar{C}}_{h}A_{h}J_{j}^{x(I)}+V_{h,j}^{\left(I\right)}\right)}^{T}\right]\left(I_{mn_{z}}-{\bar{\Lambda }}_{h}\right){\bar{\Gamma }}_{h},\;\;h\geq 1,$$

we conclude that $X_{k,h}^{\left(I\right)}=A_{k}J_{h}^{x(I)},\;h\leq k,$ and denoting:

(25) $$O_{s}^{x(I)}=\sum_{h=1}^{s}J_{h}^{x(I)}\Pi_{h}^{(I)-1}\mu_{h}^{\left(I\right)},\;\;s\geq 1;\;\;\;\;O_{0}^{x(I)}=0,$$

we have that the signal predictors and filter are given by:

(26) $${\hat{x}}_{k/s}^{\left(I\right)}=A_{k}O_{s}^{x(I)},\;\;1\leq s\leq k.$$

Secondly, to obtain the prediction and filtering estimators of the noise, ${\hat{v}}_{k/s}^{\left(I\right)},\;s\leq k$, we follow an analogous reasoning to that used for the signal estimators, which leads us to:

$$V_{k,h}^{\left(I\right)}\;=\left[D_{k}F_{h}^{T}-\left(1-\delta_{h,1}\right)\sum_{j=1}^{h-1}V_{k,j}^{\left(I\right)}\Pi_{j}^{(I)-1}{\left({\bar{C}}_{h}X_{h,j}^{\left(I\right)}+V_{h,j}^{\left(I\right)}\right)}^{T}\right]\left(I_{mn_{z}}-{\bar{\Lambda }}_{h}\right){\bar{\Gamma }}_{h},\;\;\;1\leq h\leq k,$$

and defining:

(27) $$J_{h}^{v(I)}=\left[F_{h}^{T}-\left(1-\delta_{h,1}\right)\sum_{j=1}^{h-1}J_{j}^{v(I)}\Pi_{j}^{(I)-1}{\left({\bar{C}}_{h}X_{h,j}^{\left(I\right)}+D_{h}J_{h}^{v(I)}\right)}^{T}\right]\left(I_{mn_{z}}-{\bar{\Lambda }}_{h}\right){\bar{\Gamma }}_{h},\;\;h\geq 1,$$

we obtain that $V_{k,h}^{\left(I\right)}=D_{k}J_{h}^{v(I)},\;h\leq k.$ Hence, the noise predictors and filter are given by:

(28) $${\hat{v}}_{k/s}^{\left(I\right)}=D_{k}O_{s}^{v(I)},\;1\leq s\leq k,$$

where

(29) $$O_{s}^{v(I)}=\sum_{h=1}^{k}J_{h}^{v(I)}\Pi_{h}^{(I)-1}\mu_{h}^{\left(I\right)},\;\;s\geq 1;\;\;\;\;O_{0}^{v(I)}=0.$$

Now, by substituting (26) and (28) for s=k−1 in (15), we have the following expression for the innovation:

(30) $$\mu_{k}^{\left(I\right)}=y_{k}^{\left(I\right)}-\left(I_{mn_{z}}-{\bar{\Lambda }}_{h}\right)\left({\bar{C}}_{k}A_{k}O_{k-1}^{x(I)}+D_{k}O_{k-1}^{v(I)}\right),\;\;k\geq 1.$$

Next, from (25) and (29), the following recursive relations for the vectors $O_{k}^{x(I)}$ and $O_{k}^{v(I)}$ are straightforward:

(31) $$O_{k}^{a(I)}=O_{k-1}^{a(I)}+J_{k}^{a(I)}\Pi_{k}^{(I)-1}\mu_{k}^{\left(I\right)},\;\;k\geq 1;\;\;O_{0}^{a(I)}=0,\;\;\left(a=x,v\right),$$

and from (24) and (27), it is clear that:

(32) $$\begin{array}{c} J_{k}^{x(I)}={\left({\bar{C}}_{k}B_{k}-{\bar{C}}_{k}A_{k}r_{k-1}^{x(I)}-D_{k}r_{k-1}^{vx(I)}\right)}^{T}\left(I_{mn_{z}}-{\bar{\Lambda }}_{k}\right){\bar{\Gamma }}_{k},\;\;k\geq 1,\\ J_{k}^{v(I)}={\left(F_{k}-{\bar{C}}_{k}A_{k}r_{k-1}^{xv(I)}-D_{k}r_{k-1}^{v(I)}\right)}^{T}\left(I_{mn_{z}}-{\bar{\Lambda }}_{k}\right){\bar{\Gamma }}_{k},\;\;k\geq 1, \end{array}$$

where

$$r_{k}^{ab(I)}=E\left[O_{k}^{a(I)}O_{k}^{b(I)T}\right]=\sum_{h=1}^{k}J_{h}^{a(I)}\Pi_{h}^{(I)-1}J_{h}^{b(I)T},\;\;k\geq 1;\;\;r_{0}^{ab(I)}=0,\;\;\left(a,b=x,v\right),$$

which clearly satisfies

(33) $$r_{k}^{ab(I)}=r_{k-1}^{ab(I)}+J_{k}^{a(I)}\Pi_{k}^{(I)-1}J_{k}^{b(I)T},\;\;k\geq 1;\;\;r_{0}^{ab(I)}=0,\;\;\left(a,b=x,v\right).$$

From now on, we use the following notations for simplicity:

$$O_{k}^{\left(I\right)}=\left(\begin{array}{c} O_{k}^{x(I)}\\ O_{k}^{v(I)} \end{array}\right),\;\;\;J_{k}^{\left(I\right)}=\left(\begin{array}{c} J_{k}^{x(I)}\\ J_{k}^{v(I)} \end{array}\right),\;\;\;r_{k}^{\left(I\right)}=E\left[O_{k}^{\left(I\right)}O_{k}^{(I)T}\right]=\left(\begin{array}{cc} r_{k}^{x(I)} & r_{k}^{xv(I)}\\ r_{k}^{vx(I)} & r_{k}^{v(I)} \end{array}\right).$$

*Derivation of expressions (17)–(20):*

- From (26), it is clear that ${\hat{x}}_{k/k}^{\left(I\right)}=\left(A_{k}\;,\;0\right)O_{k}^{\left(I\right)}$ and, hence, its covariance is given by $E\left[{\hat{x}}_{k/k}^{\left(i\right)}{\hat{x}}_{k/k}^{(i)T}\right]=\left(A_{k}\;,\;0\right)r_{k}^{\left(I\right)}{\left(A_{k}\;,\;0\right)}^{T}$. Then, using the OPL to write the filtering error covariances as ${\hat{\Sigma }}_{k/k}^{\left(I\right)}=E\left[x_{k}x_{k}^{T}\right]-E\left[{\hat{x}}_{k/k}^{\left(I\right)}{\hat{x}}_{k/k}^{(I)T}\right]$ and taking into account that, from *(H1)*, $E\left[x_{k}x_{k}^{T}\right]=A_{k}B_{k}^{T}$, expression (17) is proven.
- From (31)–(33), expression (18) is directly obtained.
- From (30), the expression of $\mu_{k}^{\left(I\right)}$ in (19) is straightforward.
  To obtain $\Pi_{k}^{\left(I\right)}=E\left[\mu_{k}^{\left(I\right)}\mu_{k}^{(I)T}\right]=E\left[\Gamma_{k}\left({\overset{˘}{y}}_{k}-{\hat{\overset{˘}{y}}}_{k/k-1}^{\left(I\right)}\right){\left({\overset{˘}{y}}_{k}-{\hat{\overset{˘}{y}}}_{k/k-1}^{\left(I\right)}\right)}^{T}\Gamma_{k}\right]$, we apply the Hadamar product properties and the OPL to express these matrices as:
  $$\Pi_{k}^{\left(I\right)}=K_{k}^{\gamma }\circ \left(\Sigma_{k}^{\overset{˘}{y}}-E\left[{\hat{\overset{˘}{y}}}_{k/k-1}^{\left(I\right)}{\hat{\overset{˘}{y}}}_{k/k-1}^{(I)T}\right]\right),\;k\geq 1,$$
  and using (13) for ${\hat{\overset{˘}{y}}}_{k/k-1}^{\left(I\right)}$, the expression of $\Pi_{k}^{\left(I\right)}$ in (19) is proven.
- Substituting (26) and (28) in (14), we obtain ${\hat{z}}_{k/k-1}^{\left(I\right)}=\left({\bar{C}}_{k}A_{k}\;,\;D_{k}\right)O_{k-1}^{\left(I\right)},$ and expression (20) for its covariance $\Sigma_{k}^{{\hat{z}}^{\left(I\right)}}$ is easily obtained.

*Derivation of expressions (21)–(23):*

- Expression (21) for the smoothers ${\hat{x}}_{k/k+N}^{\left(I\right)}$ is easily derived using (12), and from it, the recursive formula for the fixed-point smoothing error covariance matrices, ${\hat{\Sigma }}_{k/k+N}^{\left(I\right)}$, is immediately deduced.
- Expression (22) for $X_{k,k+N}^{\left(I\right)}=E\left[x_{k}\mu_{k+N}^{(I)T}\right]=\left(E\left[x_{k}{\overset{˘}{y}}_{k+N}^{T}\right]-E\left[x_{k}{\hat{\overset{˘}{y}}}_{k+N/k+N-1}^{(I)T}\right]\right){\bar{\Gamma }}_{k+N}$ is derived as follows:
  - On the one hand, the independence properties, together with (H1) and (8), lead us to $E\left[x_{k}{\overset{˘}{y}}_{k+N}^{T}\right]=B_{k}A_{k+N}^{T}C_{k+N}^{T}\left(I_{mn_{z}}-{\bar{\Lambda }}_{k+N}\right)$, which can be written as:
    $$E\left[x_{k}{\overset{˘}{y}}_{k+N}^{T}\right]=\left(B_{k}\;,\;0\right){\left({\bar{C}}_{k+N}A_{k+N}\;,\;D_{k+N}\right)}^{T}\left(I_{mn_{z}}-{\bar{\Lambda }}_{k+N}\right),\;\;N\geq 1.$$
  - On the other hand, using expression (13) for the one-stage predictors ${\hat{\overset{˘}{y}}}_{k+N/k+N-1}^{\left(I\right)}$ with ${\hat{z}}_{k+N/k+N-1}^{\left(I\right)}=\left({\bar{C}}_{k+N}A_{k+N}\;,\;D_{k+N}\right)O_{k+N-1}^{\left(I\right)},$ it is clear that
    $$E\left[x_{k}{\hat{\overset{˘}{y}}}_{k+N/k+N-1}^{(I)T}\right]=E\left[x_{k}O_{k+N-1}^{(I)T}\right]{\left({\bar{C}}_{k+N}A_{k+N}\;,\;D_{k+N}\right)}^{T}\left(I_{mn_{z}}-{\bar{\Lambda }}_{k+N}\right),\;\;N\geq 1.$$
  Therefore, denoting $M_{k,k+N}^{\left(I\right)}=E\left[x_{k}O_{k+N}^{(I)T}\right],\;N\geq 0$, expression (22) holds.
- Using that $O_{k+N}^{\left(I\right)}=O_{k+N-1}^{\left(I\right)}+J_{k+N}^{\left(I\right)}\Pi_{k+N}^{(I)-1}\mu_{k+N}^{\left(I\right)}$, the recursive expression (23) for the matrices $M_{k,k+N}^{\left(I\right)}$ is directly obtained. Its initial condition, $M_{k,k}^{\left(I\right)}=\left(A_{k}\;,\;0\right)r_{k}^{\left(I\right)}$, is easily derived taking into account that, from the OPL, $M_{k,k}^{\left(I\right)}=E\left[{\hat{x}}_{k/k}^{\left(I\right)}O_{k}^{(I)T}\right]$.

□

**Remark** **4.** *The simultaneous consideration of random parameter matrices, time-correlated additive noises and random deception attacks in the sensor measured outputs, together with the presence of random packet dropouts during transmission, is a novelty itself and involves some difficulties in the derivation of the proposed algorithms. One of the main challenges is concerned with the time-correlation of the noise. Since the measurement differencing approach has not been used to transform the original measurements into an equivalent set of observations that do not depend on time-related noise, a first difficulty was to obtain the noise estimators. On the one hand, since a covariance-based estimation approach is used, it has been necessary to obtain a factorization—in a separable form—for the noise covariance function (Lemma 1 (c)). On the other hand, even though the derivation of expression (28) for the noise estimators, ${\hat{v}}_{k/s}^{\left(I\right)}$, is analogous to (26) for the signal estimators, ${\hat{x}}_{k/s}^{\left(I\right)}$, additional difficulties are met when deducing simple formulas for the innovation covariances matrix, $\Pi_{k}^{\left(I\right)}$, from the innovation $\mu_{k}^{\left(I\right)}$ (expression (30)). The definition of the vectors $O_{k}^{\left(I\right)}$ and $J_{k}^{\left(I\right)}$, as well as the matrices $r_{k}^{\left(I\right)}$, made the derivation of $\Pi_{k}^{\left(I\right)}$—and, consequently, the design of the algorithms—significantly simpler. In addition, the proposed filtering and smoothing algorithms have an attractive recursive structure thanks to the above.*

### 3.3. Optimal Filtering and Fixed-Point Smoothing Algorithms (Model II)

Recursive algorithms for the centralized LS linear filter, ${\hat{x}}_{k/k}^{\left(I\;I\right)}$, and smoothers, ${\hat{x}}_{k/k+N}^{\left(I\;I\right)}$, at the fixed point *k* for any N≥1 based on the observations (10) within the compensation framework proposed in Model II, are presented in the following theorem.

**Theorem** **2.** *Under hypotheses (H1) to (H7), the centralized LS linear filtering estimators, ${\hat{x}}_{k/k}^{\left(I\;I\right)},$ and the corresponding error covariance matrices, ${\hat{\Sigma }}_{k/k}^{\left(I\;I\right)}=E\left[\left(x_{k}-{\hat{x}}_{k/k}^{\left(I\;I\right)}\right){\left(x_{k}-{\hat{x}}_{k/k}^{\left(I\;I\right)}\right)}^{T}\right]$, can be obtained by replacing the superscript “* (I) *” with “* (II) *” in (17). The vectors $O_{k}^{\left(I\;I\right)}$ and the matrices $r_{k}^{\left(I\;I\right)}$ and $J_{k}^{\left(I\;I\right)}$ are computed by replacing the superscript “* (I) *” with “* (II) *” in (18).*

*The innovations, $\mu_{k}^{\left(I\;I\right)}$, and their covariance matrices, $\Pi_{k}^{\left(I\;I\right)}=E\left[\mu_{k}^{\left(I\;I\right)}\mu_{k}^{(I\;I)T}\right]$, are obtained by:*

$$\begin{array}{c} \mu_{k}^{\left(I\;I\right)}=y_{k}^{\left(I\;I\right)}-\left(I_{mn_{z}}-{\bar{\Gamma }}_{k}{\bar{\Lambda }}_{h}\right)\left({\bar{C}}_{k}A_{k}\;,\;D_{k}\right)O_{k-1}^{\left(I\;I\right)},\;\;k\geq 1,\\ \Pi_{k}^{\left(I\;I\right)}=K_{k}^{\gamma }\circ \left(\Sigma_{k}^{\overset{˘}{y}}-\left(I_{mn_{z}}-{\bar{\Lambda }}_{k}\right)\Sigma_{k}^{{\hat{z}}^{\left(I\;I\right)}}+\Sigma_{k}^{{\hat{z}}^{\left(I\;I\right)}}{\bar{\Lambda }}_{k}\right)-{\bar{\Gamma }}_{k}{\bar{\Lambda }}_{k}\Sigma_{k}^{{\hat{z}}^{\left(I\;I\right)}}{\bar{\Lambda }}_{k}{\bar{\Gamma }}_{k},\;\;k\geq 1, \end{array}$$

*where $\Sigma_{k}^{\overset{˘}{y}}$ is given in (11) and $\Sigma_{k}^{{\hat{z}}^{\left(I\;I\right)}}$ is computed by replacing the superscript “* (I) *” with “* (II) *” in (20).*

*The recursive algorithm for the centralized LS linear fixed-point smoothers, ${\hat{x}}_{k/k+N}^{\left(I\;I\right)},$ and their error covariances, ${\hat{\Sigma }}_{k/k+N}^{\left(I\;I\right)}=E\left[\left(x_{k}-{\hat{x}}_{k/k+N}^{\left(I\;I\right)}\right){\left(x_{k}-{\hat{x}}_{k/k+N}^{\left(I\;I\right)}\right)}^{T}\right]$, is provided by (21)–(23), just replacing the superscript “* (I) *” by “* (II) *”.*

**Proof.**  The filtering and fixed-point smoothing algorithms in Theorem 2 can be proven in a similar way to those in Theorem 1; therefore, the details are omitted to save space. □

## 4. Simulation Example

In this section, we illustrate the implementation of the proposed centralized fusion filtering and fixed-point smoothing algorithms by a simulation example.

*Scalar signal process.* As in [18], let us consider a discrete-time scalar signal process ${\left\{x_{k}\right\}}_{k\geq 0}$ described by an AR(1) model. Specifically, we consider the following model (perturbed by both additive noise and signal-dependent multiplicative noise) to generate the signal:

$$x_{k+1}=\left(0.9+0.01\alpha_{k}\right)x_{k}+\varepsilon_{k},\;k\geq 0,$$

where ${\left\{\alpha_{k}\right\}}_{k\geq 0}$ and ${\left\{\varepsilon_{k}\right\}}_{k\geq 0}$ are standard Gaussian white processes and the initial signal, $x_{0}$, is a zero-mean Gaussian variable with variance 0.1. These noise sequences and the initial signal are assumed to be mutually independent; then, it is easy to establish that the signal covariance function is given by $\Sigma_{k,h}^{x}=E\left[x_{k}x_{h}\right]=0.9^{k-h}E\left[x_{h}x_{h}^{T}\right],\;h\leq k;$ hence, it is clear that it can be expressed in a separable form as $\Sigma_{k,h}^{x}=A_{k}B_{h}$, with $A_{k}=0.9^{k}$ and $B_{h}=0.9^{-h}\Sigma_{h}^{x}$, where $\Sigma_{h}^{x}=E\left[x_{h}^{2}\right]$ is recursively obtained by $\Sigma_{h}^{x}=0.8101\;\Sigma_{h-1}^{x}+1,\;h\geq 1$ with initial condition $\Sigma_{0}^{x}=0.1.$

*Sensor measured outputs.* Let us consider a three-sensor network providing scalar measurements of the signal that fit the following model:

$$z_{k}^{\left(i\right)}=C_{k}^{\left(i\right)}x_{k}+v_{k}^{\left(i\right)},\;\;\;\;k\geq 1,\;i=1,2,3.$$

In addition, let us assume, in accordance with the theoretical study, that different uncertainties are present:

- At each sensor, i=1,2,3, the random parameter sequences ${\left\{C_{k}^{\left(i\right)}\right\}}_{k\geq 1},$ are chosen to model different kinds of network-induced uncertainties, namely:
  - $C_{k}^{\left(1\right)}=0.8\theta_{k}^{\left(1\right)}$, in which ${\left\{\theta_{k}^{\left(1\right)}\right\}}_{k\geq 1}$ is a sequence of independent random variables, with uniform distribution over the interval [0.3,0.7] (continuous fading measurements in sensor 1).
  - $C_{k}^{\left(2\right)}=0.7\theta_{k}^{\left(2\right)}$, where ${\left\{\theta_{k}^{\left(2\right)}\right\}}_{k\geq 1}$ is a sequence of independent random variables with probability mass function $P\left(\theta_{k}^{\left(2\right)}=0\right)=0.1,\;\;P\left(\theta_{k}^{\left(2\right)}=0.5\right)=0.5,\;\;P\left(\gamma_{k}^{\left(2\right)}=1\right)=0.4$ (discrete fading measurements in sensor 2).
  - $C_{k}^{\left(3\right)}=0.9\theta_{k}^{\left(3\right)}$, in which ${\left\{\theta_{k}^{\left(3\right)}\right\}}_{k\geq 1}$ is a sequence of independent Bernoulli random variables with $P\left(\theta_{k}^{\left(3\right)}=1\right)={\bar{\theta }}^{\left(3\right)}$, ∀k≥1 (missing measurements in sensor 3).
  Moreover, ${\left\{\theta_{k}^{\left(i\right)}\right\}}_{k\geq 1},\;i=1,2,3,$ are assumed to be mutually independent white sequences.
- The noise processes ${\left\{v_{k}^{\left(i\right)}\right\}}_{k\geq 0}$, i=1,2,3, are defined by $v_{k}^{\left(i\right)}=D^{\left(i\right)}v_{k-1}^{\left(i\right)}+\xi_{k-1}^{\left(i\right)}$, k≥1, in which:
  - $D^{\left(1\right)}=D^{\left(3\right)}=0.8$ and $D^{\left(2\right)}=0.7$;
  - $\xi_{k}^{\left(i\right)}=a_{i}\xi_{k}$, k≥0, i=1,2,3, with $a_{1}=a_{3}=0.5$, $a_{2}=0.25$ and ${\left\{\xi_{k}\right\}}_{k\geq 0}$ a standard Gaussian white process;
  - $v_{0}^{\left(i\right)}=v_{0}$ is a standard Gaussian variable, for i=1,2,3.

In addition, according to the theoretical model under consideration, it is assumed that the measurements at each sensor are affected by deception attacks. The data injected by the attackers are described by ${\overset{˘}{z}}_{k}^{\left(i\right)}=-z_{k}^{\left(i\right)}+w_{k}^{\left(i\right)}$, in which the attack noises are defined as $w_{k}^{\left(i\right)}=w^{\left(i\right)}\zeta_{k}$, for all i=1,2,3, where $w^{\left(1\right)}=0.5$, $w^{\left(2\right)}=0.25$, $w^{\left(3\right)}=0.75$, and ${\left\{\zeta_{k}\right\}}_{k\geq 1}$ is a standard Gaussian white process. Clearly, these attack noises are correlated and $S_{k}^{\left(ij\right)}=w^{\left(i\right)}w^{\left(j\right)},\;i,j=1,2,3.$ The attacks are considered to be randomly successful or frustrated and, once the attacks are launched, the available measurements are described by (3):

$${\overset{˘}{y}}_{k}^{\left(i\right)}=z_{k}^{\left(i\right)}+\lambda_{k}^{\left(i\right)}{\overset{˘}{z}}_{k}^{\left(i\right)},\;k\geq 1,\;\;\;i=1,2,3,$$

where the Bernoulli random variables, ${\left\{\lambda_{k}^{\left(i\right)}\right\}}_{k\geq 1}$, i=1,2,3, modeling whether the deception attacks are actually successful or not, are independent and identically distributed. The probability of success is assumed to be time-invariant and to take the same value for the three sensors, namely, $P\left(\lambda_{k}^{\left(i\right)}=1\right)=\bar{\lambda },\;k\geq 1,\;i=1,2,3$.

*Observations with random packet dropouts.* The observations used for the estimation, $y_{k}^{\left(i\right)}$, are described using one of the following two models:

(34) $$\begin{array}{c} \;Model\;I\;⟶\;y_{k}^{(i)I}=\gamma_{k}^{\left(i\right)}{\overset{˘}{y}}_{k}^{\left(i\right)}+\left(1-\gamma_{k}^{\left(i\right)}\right){\hat{\overset{˘}{y}}}_{k/k-1}^{(i)I},\;\;k\geq 1,\;\;\;i=1,2,3,\\ Model\;II\;⟶\;y_{k}^{(i)I\;I}=\gamma_{k}^{\left(i\right)}{\overset{˘}{y}}_{k}^{\left(i\right)}+\left(1-\gamma_{k}^{\left(i\right)}\right){\hat{z}}_{k/k-1}^{(i)I\;I},\;\;k\geq 1,\;\;\;i=1,2,3, \end{array}$$

where the sequences modeling the transmission packet losses, ${\left\{\gamma_{k}^{\left(i\right)}\right\}}_{k\geq 1}$, i=1,2,3, are independent sequences of independent Bernoulli random variables with time-invariant packet arrival probabilities, which are the same for the three sensors, $P\left(\gamma_{k}^{\left(i\right)}=1\right)=\bar{\gamma },\;\forall k\geq 1,\;i=1,2,3$.

A MATLAB program has been developed to obtain the centralized fusion estimators and the corresponding error variances, and fifty iterations of the estimation algorithms proposed in Theorems 1 and 2 have been run for the above observation models with random packet dropouts. The estimation accuracy has been analyzed by examining the error variances for several probabilities ${\bar{\theta }}^{\left(3\right)}$ of the Bernoulli random variables modeling the missing measurement phenomenon of the third sensor and different values of $\bar{\lambda }$ and $\bar{\gamma }$ that determine the probabilities of successful attacks and packet dropouts during the process of data transmissions, respectively.

**Figure 2:**
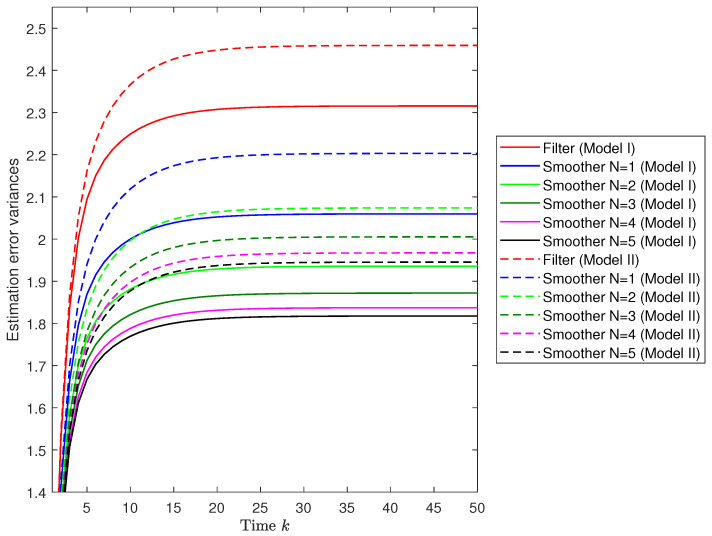
Error variance comparison of the centralized fusion filtering and smoothing estimators considering observations from Model I and Model II.

*Performance of the centralized fusion filtering and fixed-point smoothing estimators.* Let us assume the same value, 0.5, for the probabilities ${\bar{\theta }}^{\left(3\right)},$$\bar{\lambda }$ and $\bar{\gamma }$. First, the error variances are analyzed to compare the proposed filtering and fixed-point smoothing estimators considering both observation models—Model I and Model II— given in (34). The results of this comparison are displayed in Figure 2, which shows, on the one hand, that both estimators (filter and smoother) present lower error variances under Model I than under Model II. On the other hand, it is gathered that, for both observation models, the smoothing error variances are less than the filtering ones. Additionally, it can be inferred that as the number of available observations increases, the smoothers at each fixed-point *k* become more accurate; this fact is more pronounced for N≤5, since the difference is practically negligible for N>5.

**Figure 3:**
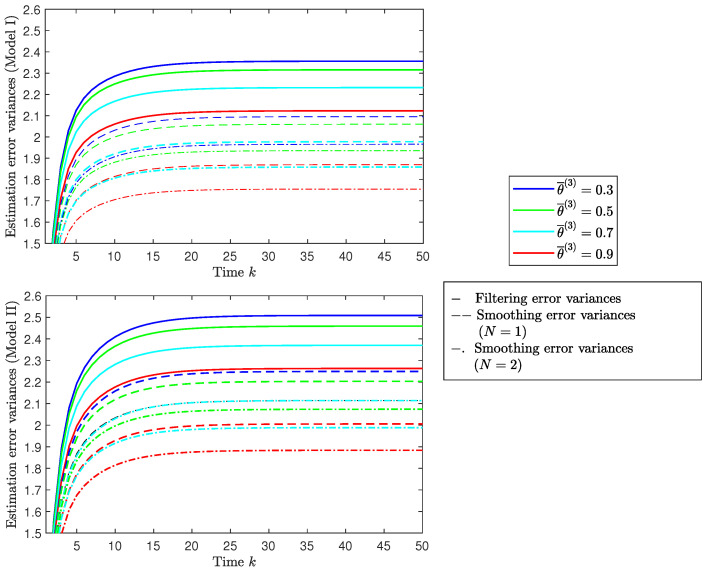
Centralized fusion filtering and smoothing error variances for ${\bar{\theta }}^{\left(3\right)}\;\;=0.3,0.5,0.7,0.9$ under Model I and Model II.

*Influence of the missing measurement probabilities (sensor 3).* Let us assume that, as in Figure 2, the attack and packet arrival probabilities are $\bar{\lambda }=0.5$ and $\bar{\gamma }=0.5$, respectively. Figure 3 displays the filtering and smoothing error variances for both models, considering several values of the probability ${\bar{\theta }}^{\left(3\right)}$ (more precisely, ${\bar{\theta }}^{\left(3\right)}=0.3,0.5,0.7$ and 0.9) to illustrate the impact of the missing measurement phenomenon in sensor 3. For both models, the error variances show a similar behavior, and we can draw the conclusion that the probability ${\bar{\theta }}^{\left(3\right)}$ that the signal is present in the measured outputs of sensor 3 indeed has an effect on the estimators’ performance. Actually, as it could be predicted, the estimation error variances drop as the values of this probability rise; as a result, the filtering and smoothing estimators perform better when the probability of missing data, $1-{\bar{\theta }}^{\left(3\right)}$, decreases. As in Figure 2, this figure also shows that the error variances corresponding to the smoothers for both models are lower than those of the filters and that the smoother with lag N=3 performs better than the smoother with lag N=1.

**Figure 4:**
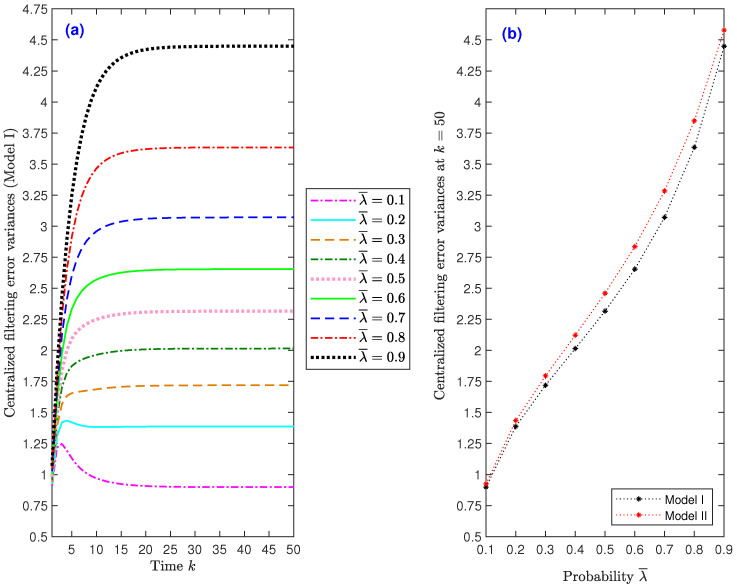
Centralized fusion filtering error variances: (**a**) under Model I when $\bar{\lambda }=0.1$ to 0.9; (**b**) under Model I and Model II at k=50 versus $\bar{\lambda }$.

*Effect of the successful deception attack probabilities.* We examine the impact of the deception attacks on the estimation accuracy considering, as in Figure 2, ${\bar{\theta }}^{\left(3\right)}=0.5$ and $\bar{\gamma }=0.5$. For this purpose, we compare the filtering error variances for several values of the successful attack probability of the three sensors, $\bar{\lambda }$. Under Model I, Figure 4a shows the filtering error variances for $\bar{\lambda }\;=0.1$ to 0.9; it can be seen from this figure that the filter performance is indeed affected by this probability, showing—as expected—a deterioration when the attack probability, $\bar{\lambda }$, rises (similar results are obtained under Model II). Taking into account that, from k=25 onwards, the behavior of the error variances is analogous in all the iterations, Figure 4b only displays the filtering error variances at iteration k=50, versus $\bar{\lambda }$, to better illustrate this decreasing trend for both Model I and Model II. Similar outcomes, and hence the same conclusions, are drawn for the smoothing error variances.

**Figure 5:**
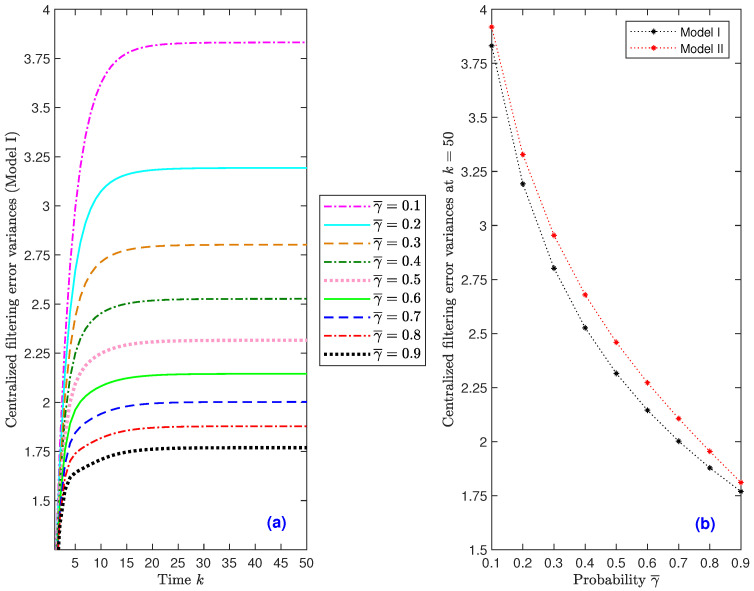
Centralized fusion filtering error variances: (**a**) under Model I when $\bar{\gamma }=0.1$ to 0.9; (**b**) under Model I and Model II at k=50, versus $\bar{\gamma }$.

*Influence of the transmission loss probabilities.* For ${\bar{\theta }}^{\left(3\right)}=0.5$ and $\bar{\lambda }=0.5$, different values for the probability $\bar{\gamma }$ of the Bernoulli variables modeling the packet loss phenomenon have been considered to analyze their influence on the filter performance. Under Model I, Figure 5a compares the filtering error variances for $\bar{\gamma }$ ranging from 0.1 to 0.9. This figure leads to the conclusion that the error variances decrease as $\bar{\gamma }$ increases, meaning that, as expected, better estimations are obtained when the probability of packet dropouts during transmission decreases. Similar results—and, consequently, analogous conclusions—are obtained in all the considered scenarios when assuming different packet arrival probabilities and comparing the error variances for Model II. As in Figure 4b, Figure 5b only displays the filtering error variances at iteration k=50, versus $\bar{\gamma }$, to better visualize the decreasing trend of such error variances under both Model I and Model II. Similar outcomes, and hence the same conclusions, are drawn for the smoothing error variances.

## 5. Conclusions

Recursive algorithms for the centralized fusion optimal linear filtering and fixed-point smoothing estimation problems are proposed from multi-sensor measurements perturbed by random parameter matrices, time-correlated additive noises and random deception attacks. Under the assumption that some data packets may be randomly lost during the transmission process from the sensors to the processing center, two compensation scenarios—both on the basis of the prediction compensation methodology—are analyzed. The first one consists of using the prediction estimator of the lost measurement (i.e., the one transmitted by the sensor after the attack is launched) as a compensator, whereas the second one uses the prediction estimator of the actual output measured by the sensor before the attack is launched. Both scenarios are compared by some numerical results, which show the performance of the proposed estimators and illustrates how the theoretical system model under consideration covers some common networked-induced phenomena (namely, missing measurements and fading measurements). Furthermore, the influence of the missing measurement probabilities, the effect of the deception attack success probabilities and the impact of the transmission dropout probabilities on the estimation accuracy are also analyzed in the context of the numerical results.

An interesting task to be considered in the future would be the theoretical study of the influence of the successful attack probabilities and the transmission loss probabilities on the estimation accuracy. In addition, the theoretical and experimental comparison between the proposed compensation models and some other compensation schemes—such as the zero-input or the hold-input schemes—could be considered. The design of quadratic or polynomial estimation algorithms that outperform the widely used linear ones is also an interesting further research topic.

## Data Availability

Not applicable.
